# Topic-based classification and identification of global trends for startup companies

**DOI:** 10.1007/s11187-022-00609-6

**Published:** 2022-03-01

**Authors:** Ivan Savin, Kristina Chukavina, Andrey Pushkarev

**Affiliations:** 1 Institute of Environmental Science and Technology, Universitat Autònoma de Barcelona, Barcelona, Spain; 2 Graduate School of Economics and Management, Ural Federal University, Yekaterinburg, Russian Federation

**Keywords:** Crunchbase, Machine learning, Natural language processing, Investments, Entrepreneurship, M13, C6, F23, L26

## Abstract

To foresee global economic trends, one needs to understand the present startup companies that soon may become new market leaders. In this paper, we explore textual descriptions of more than 250 thousand startups in the Crunchbase database. We analyze the 2009–2019 period by using topic modeling. We propose a novel classification of startup companies free from expert bias that contains 38 topics and quantifies the weight of each of these topics for all the startups. Taking the year of establishment and geographical location of the startups into account, we measure which topics were increasing or decreasing their share over time, and which of them were predominantly present in Europe, North America, or other regions. We find that the share of startups focused on data analytics, social platforms, and financial transfers, and time management has risen, while an opposite trend is observed for mobile gaming, online news, and online social networks as well as legal and professional services. We also identify strong regional differences in topic distribution, suggesting certain concentration of the startups. For example, sustainable agriculture is presented stronger in South America and Africa, while pharmaceutics, in North America and Europe. Furthermore, we explore which pairs of topics tend to co-occur more often together, quantify how multisectoral the startups are, and which startup classes attract more investments. Finally, we compare our classification to the one existing in the Crunchbase database, demonstrating how we improve it.

## Abstract (plain language summary)

We propose a novel classification of more than 250 thousand startups registered in the Crunchbase database based on machine learning algorithms and free from expert bias. We find that the share of startups focused on data analytics, social platforms, and financial transfers, and time management has risen, while an opposite trend is observed for mobile gaming, online news, and online social networks as well as legal and professional services. We also identify strong regional differences in class distribution, suggesting, for example, sustainable agriculture being present stronger in South America and Africa, while pharmaceutics, in North America and Europe. Our classification can improve analysis of the Crunchbase data further promoting the popularity of the platform, while the trends identified will be useful for investors and policy makers. Last not least, this paper presents the first application of topic modeling to startup companies, providing thus a new direction for academic research.

## Introduction

According to the Global Startup Ecosystem Report (Global Entrepreneurship Network, 2020), the global startup economy continues to grow, producing around 3 trillion USD in value for the period from 2017 to the first half of 2019, which is comparable with the GDP of Germany or France. The Crunchbase funding report (Rowley, 2020) demonstrates that private market investment grew substantially, and projections show that 1.5 trillion USD over the past decade was globally invested in venture capital deals. According to the study by Florida and Hathaway (2018), there is an extensive increase in startup and venture capital activity since 2009. Globalization processes and the new era of technological innovation rapidly change the geography of startup activity around the world: now not only the USA, but all regions are present on the startup and venture capital investment map. Menon (2018) claims that the venture capital industry evolves rapidly, and traditional data sources could not cover all the current trends. For instance, in 2015, Chinese companies operating in the field of artificial intelligence were not known abroad, whereas only 2 years later, the Chinese market was the second largest global player after the USA. Startups are an essential part of the economy. These companies develop new ideas and technologies that can drastically change established markets and industries. Companies like Apple, Microsoft, and Tesla have once all been startups. Therefore, it is important to understand what startup entrepreneurs are focused on and how this focus changes over time.

This study aims to explore global trends among startup companies using textual descriptions from the Crunchbase database. We analyze more than 250 thousand startup companies in the period 2009–2019. Since the dataset presents a large volume of information that can hardly be analyzed and classified manually, we employ the topic modeling method. This is a computer-based approach developed at the intersection of machine learning and natural language processing that allows to discover distinct topics presented in text. The advantage of topic modeling is that it avoids potential inconsistency arising from subjective assessments by human coders, requires little time to analyze the data, and assures reproducibility. In particular, we use structural topic modeling (STM, Roberts et al., 2014), which is able to utilize additional information about the texts. In our case, we take into account the geographical location of the startups’ headquarters and the year of establishment. Compared to alternative topic modeling techniques, STM has been found to generate better topics for relatively short texts as is common for descriptions in the Crunchbase database (Roberts et al., 2014).

By eliciting common topics in those descriptions, we propose a novel and objective classification of startup companies. It consists of 38 classes (“topics”) where we measure the prevalence (weight) of each of these classes for each of the startups. Considering the year of establishment of the startups, we observe the positive time trends for data analytics and artificial intelligence, time management, social platforms, financial transfers, and cryptocurrency, and negative time trends for mobile gaming, online social networks, search engine optimization and marketing services, online news and blogs, and legal and professional services. Geographic concentration of startup activity shows that depending on the region where the startup has been established, the prevailing classes are different. For instance, North American startups tend to focus on the fields related to information technology, medicine, and finance, similar to European startups where we find higher prevalence of IT, pharmaceutics, transport, and logistics. The Asian market generates proportionally more startups in science and tech services, data analytics and AI, and legal and professional services.

We also explore which pairs of topics tend to occur more often together illustrating the multidisciplinary nature of the startups which is slightly reducing over time. Furthermore, we find that some topics are strongly overlapping in the same startup descriptions. One of the brightest examples is an overlap of financial, transfer, and science and technology services, also known as fintech. Block et al. (2018) discuss emergence of fintech startup companies arguing that technological progress and new social media together with crowdfunding and changes in IPO regulation stimulated their development. Today, fintech companies pose a considerable threat to traditional actors on financial markets (such as banks), demonstrating how startups combining different classes can be more lucrative for entrepreneurs and investors. In Sect. 4, we discuss more examples of such multidisciplinary startups.

Furthermore, we study how the shares of startup classes change after we take the amount of investments they collected into account. Among others, we find that classes related to sustainable agriculture and pharmaceutics considerably increase their share. The fact that the class on sustainable agriculture is so important further supports earlier argument by Van Gelderen et al. (2021) that “entrepreneurship will become more necessity focused […] addressing social and environmental challenges in a local manner.”

Finally, we illustrate that the STM classification compared to the one from Crunchbase provides better distinguishable and more concrete classes of more even size enabling their better comparison and helps to avoid expert bias (i.e., the situation when startup founders attribute their companies to too few or too many classes in the Crunchbase classification).

Our findings are valuable for several reasons. First, as we show in the paper, the Crunchbase database is actively used in academic research, but also well beyond by other stakeholders. A more accurate and reliable classification will contribute to better analysis of the data further promoting the popularity of the platform. Second, startup founders, investors, and policy makers will find our results on the trends on startup classes across time and space useful for their decisions on where to locate companies, where to invest, or whom to invite for collaboration. Third, our work contains many empirical findings that we connect to the previous literature on entrepreneurship, sometimes supporting earlier results with new evidence, and sometimes questioning the conclusions drawn earlier. Last not least, this paper presents the first application of topic modeling to startup companies, providing thus a new direction for academic research.

The remainder of this paper is organized as follows. Section 2 provides some background literature on trends and classifications of startup companies, and on the application of topic modeling in economic literature. Section 3 describes our data and methods. Section 4 presents the results on the topic-based classification identified by STM and its comparison with the Crunchbase classification, startup multidisciplinarity in the STM classification, differences among topic prevalences across time and space, and their heterogeneity regarding the investments received. Section 5 concludes.

## Literature review

### Trends in startup classification

Concerning data sources on startup companies, Crunchbase is becoming increasingly prevailing. More than 100 scientific contributions based on its data have been published already by 2017 (Dalle et al., 2017). For instance, Block and Sandner (2011) analyzed the state of the venture capital market in 2010–early 2011 and concluded that Crunchbase data is representative for data on venture capital from other data sources including the US National Venture Capital Association. Hunter et al. (2018) constructed a dynamic network of investors and companies to evaluate the quality of startup companies. Ratzinger et al. (2018) examined the role of higher education of digital startup founders on financial performance of their companies, demonstrating its significant and positive influence. Haddad and Hornuf (2018) use Crunchbase data to analyze emergence of fintech startups related to a range of country-level indicators like availability of venture capital, quality of Internet servers, and ease of access to loans. Recent study by Żbikowski and Antosiuk (2021) develops predictive models based on machine learning and the Crunchbase data to forecast a company’s success.

To the best of our knowledge, the literature dedicated to classification of startups is rather limited. A recent study by Felgueiras et al. (2020) used the Crunchbase data and machine learning techniques to predict classification of new startup descriptions achieving 70% precision with their approach. The authors use the existing Crunchbase classification that, as they confirm, is very unbalanced. We, on the other hand, suggest that the Crunchbase classification has too many limitations and offer an alternative classification of the startups.

Other research in the area of startup classification is mainly focused on certain sectors. For example, Teuteberg et al. (2018) focus on startups in the financial sector that use blockchain. They propose seven classes with trading platforms being the largest. Chakraborty et al. (2021) review 76 scientific articles devoted to health-related startups and identify five classes (“themes”): electronic health services, technology adoption, business planning and framework, psychographics, and regulations. Palmié et al. (2021) concentrate on 280 startups and incumbents from the electricity sector providing a classification of business models. While all these studies are focused on a subset of companies working in a particular economic sector, we aim to produce a general classification of all startups providing a comprehensive overview of technology-related companies worldwide.

### Topic modeling and its application for classification

Traditionally, firms are attributed to certain classes in large databases either by human coders (administrators of the database) or, as is the case for the Crunchbase database, the startup founders themselves. This has the disadvantage that the classification is incomplete (i.e., some founders simply do not fill out this field by entering information about their company) and subject to expert bias, i.e., when a person classifies a company to too many or too few classes, “industry groups” as they are called in Crunchbase (see examples in Sect. 4). As a result, the classification is also inconsistent as not the same scale is applied to all the companies. Furthermore, new classes in such big datasets are typically introduced with a considerable time delay. All these limitations can lead to misleading conclusions about the startup companies. Users of the Crunchbase database may find too few or too many startups in the area of their interest, and have wrong expectations about the trends in a certain startup class.

With the rising power of computers and machine learning algorithms, however, our choice of instruments to extract information from the textual data and classify it has considerably increased. In this study, we apply the so-called topic modeling (TM) approach to elicit topics from companies’ descriptions and classify them according to these topics. TM is a clustering approach for textual data aimed to identify meaningful topics in texts, analyze trends in topics, and (re)classify and annotate documents (Blei, 2012).

Earlier topic modeling has been applied to different types of textual information in social sciences and in economics in particular. Many studies focus on scientific literature published either in specific peer-reviewed journals across many themes (Griffiths & Steyvers, 2004; Lüdering & Winker, 2016; De Battisti et al., 2015; Chen et al., 2020: Savin & van den Bergh, 2021), all economic (in total over 250 thousand) articles stored in a given database (Ambrosino et al., 2018) or all scientific articles about climate change (400 thousand articles, see Callaghan et al., 2020). Another popular field of application for TM is news articles, reports, and posts in social networks (Chae & Park, 2018; Huang et al., 2017; Jacobi et al., 2016; Kim & Ju, 2019). For example, Larsen et al. (2019) analyze news from the Norwegian business newspaper, and link obtained topics and their prevalence to the economic fluctuations on the asset markets. TM has recently been applied also to patent data in order to (i) (re)classify those into product and technology sub-classes and later explore technological convergence for the photovoltaic technology in the USA (Venugopalan & Rai, 2015); (ii) identify emerging topics in the USA, EU, and Japan (Lee et al., 2015); (iii) detect pioneering patent introducing new topics (Kaplan & Vakili, 2015); and (iv) predict trends in patent topics (Chen et al., 2017; Choi & Song, 2018; Savin et al., 2022; Suominen et al., 2017). TM has been further applied to survey open-ended questions to examine public perceptions of economic growth (Savin et al., 2021) and climate change (Tvinnereim & Fløttum, 2015; Tvinnereim, Liu, et al., 2017), to collect ideas on what people think about climate change mitigation measures in general (Tvinnereim, Fløttum, et al., 2017) and carbon pricing as a policy instrument in particular (Savin et al., 2020), or about other individuals’ beliefs about climate change (Mildenberger & Tingley, 2017).

All these examples illustrate that TM can be applied to very different types of textual data in terms of size and content. TM can efficiently classify texts into topics, and demonstrate dynamics of those topics and their mutual relation. Thanks to TM application, many new trends and patterns have been observed, which otherwise would be very hard to impossible to make using human coders. TM thus represents a powerful tool from the field of artificial intelligence that can deal with very large amount of data and provides a comprehensive picture about thousands of companies, patents, or studies in contrast to only hundreds of startups and dozens of studies that have been analyzed manually (see examples in Sect. 2.1). It clearly represents a step forward in dealing with textual data, which is confirmed by its massive application over the last 10 years to so many different domains of knowledge. Nonetheless, we are not aware of TM being applied to startup data. Therefore, this study also presents a novel application of the method in the literature.

## Data and methods

### Data

We use data from Crunchbase, the leading platform for professionals to discover innovative companies (https://www.crunchbase.com/). Crunchbase has more than 55 million yearly active users, 4 thousand venture program members, and 1.3 billion API calls yearly.^1^ Crunchbase sources its data in four ways: the venture program, machine learning algorithms, an in-house data team, and the Crunchbase community. New submissions to register startup companies in the database are subject to registration and social validation, and are reviewed by a moderator before being accepted for publication. The Crunchbase team also moderates existing profiles and removes irrelevant or spammy content on a daily basis.

Following the recent literature regarding Crunchbase as the “premier sources of venture capital data” (Bellavitis et al., 2021) that reports information on “entrepreneurial actors” (Alaassar et al., 2021) “active in a particular year and country” (Haddad & Hornuf, 2019), we consider all companies at the year of their foundation published in the Crunchbase database as active startup companies. For example, Tinder registered in Crunchbase with a year of establishment being 2012 was a startup in that particular year. The coverage of the database spans from firms that became very large in the recent past (Cojoianu et al., 2021) to small companies that might have been founded recently (Alexy et al., 2012). Crunchbase predominantly contains ambitious “venture capital oriented innovative entrepreneurial firms” (Leendertse et al., 2021) “as opposed to restaurants, nail salons, and other more personal, life-style, and conventional entrepreneurship” (Yu & Fleming, 2021).

Studying all the startups registered in Crunchbase over the period 2009–2019 allows us, for the first time, to provide a novel startup classification free from previous shortcomings and highlight important trends regarding evolution of those classes over time, their spatial concentration, and concentration of investments among those classes.

Crunchbase provides its own classification of industry classes (Crunchbase classification, henceforth), based on industry categories of companies that have been added by the Crunchbase team, investors, the companies themselves, or any of the platform contributors.^2^ Overall, there are 46 classes, including, for example, information technology, real estate, agriculture and farming, and biotechnology. Startups can be attributed simultaneously to more than one class. Distribution of startups across these classes is very uneven ranging from less than 1% for Government and Military to almost 38% for Software (for more details, see Fig. 11 in Appendix 1). Furthermore, 3.2% of startups (around 8,000 companies in our sample) have no class attribution. Based on the share of class assignments in the number of startups established in specific years, one can observe positive trends for classes like “Software,” “Information Technology,” “Financial Services,” “Data and Analytics,” and “Science and Engineering,” and negative trends for “Internet Services,” “Media and Entertainment,” “Sales and Marketing,” “Design,” and “Advertising.”

We collected data from the Crunchbase website in May 2020. The dataset represents information on 366,274 startups founded in the period between 2009 and 2019 around the world. The period before 2009 is not included in our analysis, since the rapid development and geographic expansion of startups and venture capital activity have started in 2009 together with accelerated globalization processes and technological development around the world (Florida & Hathaway, 2018). The year 2020 was not included to not distort our analysis by the recent COVID-19 crisis.

According to the database, startup companies tend to establish headquarters in North America (40.5% of all startups), Europe (24.7%), and Asia (16.3%). Around 12.1% of all startups have not identified headquarter locations of their businesses.

**Fig. 1:**
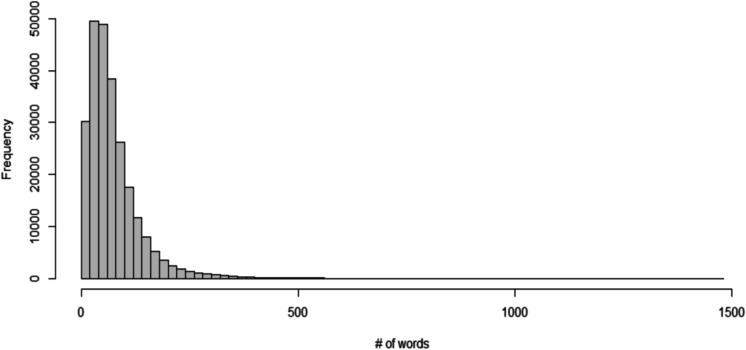
Length of full startup descriptions. Note: on the X-axis, the shortest length of response is 1, and the longest, 1464

In this study, we are focusing our analysis on the companies’ full descriptions, which is available for 254,055 startups in our sample. Unlike short descriptions that consist of a single sentence and are no longer than 32 words only, full descriptions have a mean (median) length of 75 (59) words with a small fraction of texts well exceeding 100 words (see Fig. 1 below).

Doing an initial screening of these textual descriptions, we found that not all of them are equally informative: some did not display information of the main company activity, but only contact information or location, while some other descriptions were not in English. These descriptions were deleted from further analysis marginally reducing our sample to 250,252 startups. Removing these observations from the dataset does not considerably change the distribution of startups across countries and years of establishment (see Tables 2 and 3 in Appendix 1).

It is worth mentioning that textual descriptions of startups have been already analyzed in the recent literature, even though not by means of the topic modeling approach. For example, Bollaert et al. (2020) use textual descriptions of the crowdfunding projects to classify the projects by the level of the founders’ narcissism. They show that more narcissistic entrepreneurs set less ambitious goals and are less successful than their peers. Kaminski and Hopp (2020) use text, speech, and video metadata about crowdfunding campaigns and predict their outcomes by means of a combination of neural network and paragraph vector approach. Their findings highlight the importance of descriptions’ certainty regarding the state of product development and the product itself for its success. Thus, both studies show that textual data can be useful for determining characteristics of the startups and provide further insights about their success.

### Methodology and data pre-processing

To elicit hidden (latent) topics in the startup textual descriptions, we use the topic modeling (TM) method. Essentially, it is a clustering algorithm organizing startup descriptions in different topics. Topic modeling “groups” words into topics based on the co-occurrence of words in the startup descriptions and then assesses the weight of each topic in each individual description. For example, if we see the word “blockchain” in a topic labeled “Financial services,” we can understand that it appears frequently in combination with other words of this topic, meaning that blockchain technology is mostly employed in the topic of financial instruments and transfers.

Formally speaking, TM makes a Bayesian inference of words related to a given topic and the topics being discussed in a given company description, based on descriptions already observed. In particular, TM assumes that each word in the descriptions is generated through a two-step process: first, each startup description has its own distribution of topics, and a topic is randomly drawn from it; second, each topic has its own word distribution, and a word is randomly drawn from this distribution for the topic selected in the first step. Essentially, each description is a result of repeating these two steps many times. Therefore, the startup descriptions may have multiple topics present in them in different proportions. Topic modeling discovers the topic distribution for each description and the word distribution of each topic iteratively, by fitting this two-step procedure to the observed descriptions until it finds the best model that describes the underlying data. Compared to simple count of keywords, TM considers words not in isolation, but accounting for their context, which can influence the meaning of the words.

An important advantage of structural topic modeling (STM) over classical TM is that it can take into account additional information about the texts. In our case, we consider the location of the startups (North America, EU, Asia, Africa, South America, Australia, and Oceania^3^) and the year it was founded. Using additional data as covariates at the stage of estimating a topic model has proven to result in higher quality topics (Roberts et al., 2014). After forming a topic model, we can later use these covariates to understand dynamics in the popularity of different topics and their geographical distribution (see Sect. 4).

Before applying STM on the textual descriptions, we need to make some standard pre-processing steps. First of all, capital letters have been converted to a lower case; special characters, accents, stop words (like pronouns, prepositions, and other common words), and words shorter than three letters were removed. Numbers and special characters were excluded. After that, all words have been lemmatized, i.e., converted to their vocabulary form (e.g., “walking” is converted to “walk”). To reduce noise in the data, we have also removed words that appear in less than ten textual descriptions. The last step is typical for topic modeling as rare words are hard to classify in any topic due to lack of observations. After this procedure, we are left with 250,226 startup descriptions, 16,917 unique words, and 7,073,171 words occurring with repetition.

**Fig. 2:**
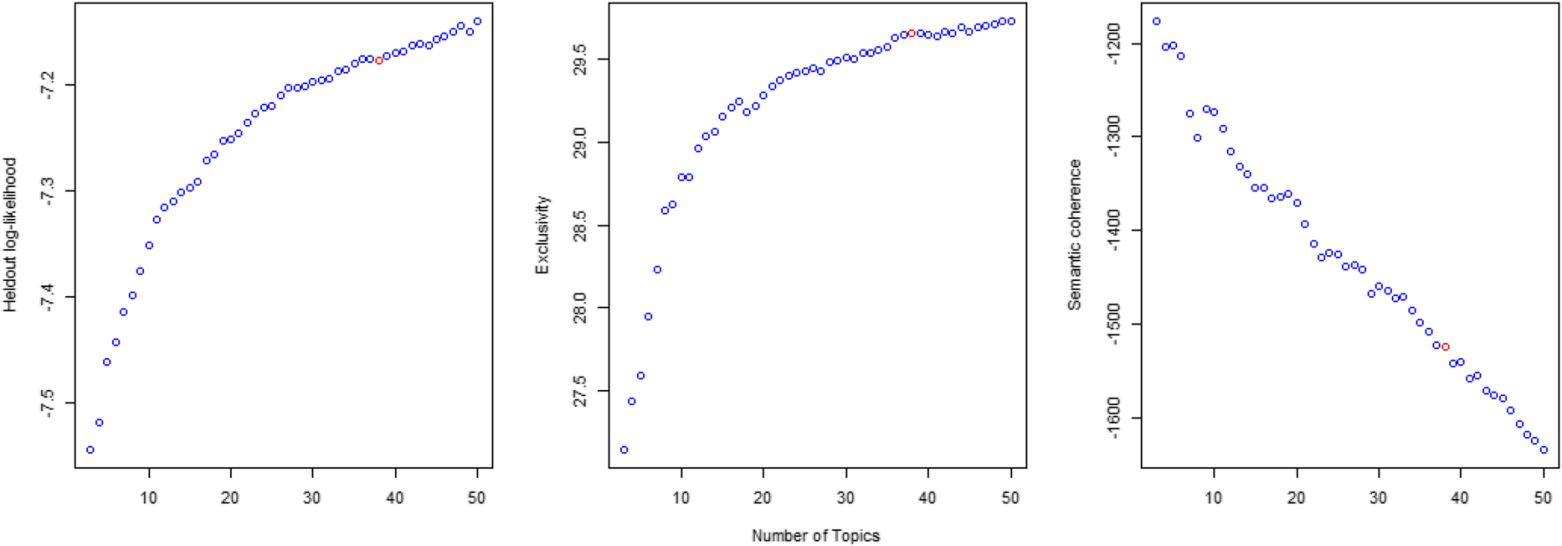
Model performance for distinct number of topics for the startup descriptions

To apply STM, we use the associated R package developed by Roberts et al. (2019). As it is common for clustering methods, the number of topics *k* has to be determined first. The method then assigns to each topic a vector with *k* weights (“topic prevalences”) for each startup description. It, essentially, shows the degree to which the description relates to each topic. Those weights sum up to 1. If the description belongs to one topic exclusively, all weights, except one, will be zeros. This, however, is rarely the case. We also estimate topic prevalence for the whole dataset to measure topic weights, which represent the share of each topic (class) among all startup companies (see Table 1). To define the number of topics *k*, we consider topic model performance on three criteria, namely “heldout log-likelihood” of the models (the accuracy of the model to predict word distribution from a sample that has been excluded from the estimation step), exclusivity (the degree to which popular words from different topics overlap), and semantic coherence (how frequently words from the same topic co-occur within each response). Figure 2 shows the performance of alternative model specifications (*k* ranges from 3 to 50^4^). Selecting the optimal number of topics is challenging, particularly since coherence tends to fall with the number of topics, while the prediction accuracy and exclusivity, in contrast, tend to rise. Next to prediction accuracy, exclusivity, and coherence, a fourth implicit consideration in selecting a topic number is the model’s complexity (number of topics): the larger it is, the harder it is to interpret the results. On this basis, we select the number of topics to be 38, as highlighted in red color in Fig. 2. Exclusivity for this number of topics reaches a local maximum and is only marginally lower than for the models with over 40 topics, while potential loss in prediction accuracy is compensated by lower model complexity and maintained coherence.

**Table 1:** Topics identified for startup descriptions

№	Topic label	Most discriminating terms (in terms of frequency and exclusivity)	Topic prevalence
1	Wellness	Sleep, dental, mental, stress, anxiety, addiction, yoga, emotional, habit, wellbeing	1.0%
2	Travel and tourism	Rental, trip, traveler, apartment, vacation, travel, hotel, taxi, accommodation, tourist	2.7%
3	Data analytics and AI	Intelligence, analytics, insight, algorithm, analyze, actionable, analysis, artificial, edge, datum	4.4%
4	Graphic design	Photographer, art, gallery, photo, interior, artwork, photography, upload, snap, renovation	1.1%
5	Time management	Time, task, real, track, spend, automatically, dashboard, email, report, feedback	4.7%
6	Healthcare services	Healthcare, pet, doctor, pharmacy, nurse, caregiver, medication, clinic, hospital, practitioner	1.4%
7	Online education	Teacher, tutor, lesson, parent, school, classroom, educational, educator, teach, learner	2.3%
8	Fitness	Train, coach, trainer, gym, workout, golf, athlete, goal, force, motivate	1.8%
9	Trash (location, time of establishment)	Japan, Beijing, Shanghai, Tel Aviv, China, Tokyo, stealth, Israel, Finland	1.6%
10	Energy	Energy, solar, electricity, renewable, grid, cbd, gas, hemp, fuel, turbine	1.0%
11	Clothes and accessories	Shoe, dress, fashion, clothe, apparel, jewelry, footwear, outfit, shirt, gift	2.5%
12	Science and technology services (t-KIBS)	Engineer, innovation, field, technological, technical, expertise, innovative, swiss, excellence, tech	4.2%
13	Food and beverages	Coffee, wine, recipe, beer, chef, tea, delicious, snack, brew, cook	2.0%
14	Transport and logistics	Drone, aerial, satellite, autonomous, inspection, aircraft, unman, uav, building, weather	2.3%
15	Recruitment services	Job, recruitment, career, recruit, hire, employee, resume, recruiter, talent, seeker	2.6%
16	Supply and distribution	Supplier, chain, cannabis, battery, supply, bicycle, cigarette, roof, procurement, light	1.5%
17	Social platforms	Idea, passionate, passion, thing, put, great, hard, start, dream, hope	6.5%
18	Financial transfers and cryptocurrency	Loan, currency, crypto, cryptocurrency, bitcoin, credit, blockchain, payment, token, lend	3.3%
19	Cybersecurity	Cyber, security, cybersecurity, server, authentication, document, encryption, password, threat, encrypt	2.7%
20	Medical devices	Surgical, ultrasound, surgery, procedure, minimally, surgeon, invasive, cardiac, stroke, brain	1.1%
21	Telecommunication devices and services	Voice, sound, bluetooth, wireless, wifi, call, laptop, noise, plug, keyboard	1.7%
22	Software development	Software, gile, integration, saas, erp, salesforce, management, implementation, automation, suite	4.4%
23	Sustainable agriculture	Farmer, farm, agricultural, sustainable, waste, water, sustainability, soil, planet, eco	1.2%
24	Investment management	Investment, capital, venture, advisory, investor, equity, advisor, growth, strategic, invest	4.6%
25	Mobile gaming	IOS, android, game, iPhone, app, mobile, ipad, gaming, multiplayer, blackberry	2.0%
26	Augmented and Virtual reality	Reality, augment, virtual, smart, home, assistant, immersive, touch, presentation, interaction	1.3%
27	Manufacturing	Printer, metal, steel, composite, manufacture, mold, fiber, factory, coating, wood	2.1%
28	Event management	Event, venue, organizer, fan, concert, musician, league, music, attendee, football	2.1%
29	Video and animation	Video, creator, content, animation, publisher, storytelling, audience, youtube, viewer, multimedia	2.4%
30	Trash (location)	San Francisco, Brazil, California, Sao Paulo, nonprofit, unite, Latin, Chicago	3.8%
31	E-commerce	Seller, coupon, discount, buyer, deal, price, purchase, retailer, auction, sell	4.1%
32	Online social networks	Twitter, influencer, facebook, share, friend, chat, social, follower, user, conversation	4.2%
33	SEO and online marketing services	SEO, campaign, agency, engine, advertise, wordpress, marketer, web, digital, advertiser	4.2%
34	Online news and blogs	News, review, article, www, reader, blog, http, answer, https, write	3.4%
35	Pharmaceutics	Therapeutics, biotechnology, genetic, biotech, molecule, gene, antibody, molecular, biopharmaceutical, genomics	2.0%
36	Beauty and cosmetics	Hair, salon, makeup, skincare, nail, beauty, tattoo, cosmetic, spa, fragrance	0.5%
37	Legal and professional services (p-KIBS)	Legal, lawyer, service, Australia, attorney, law, Delhi, Bangalore, India, pvt	4.3%
38	Parking	Space, park, owner, spot, demand, revolutionize, zone, garage, gate, intend	1.0%

## Results and discussion

### Topic-based classification identified by STM

**Fig. 3:**
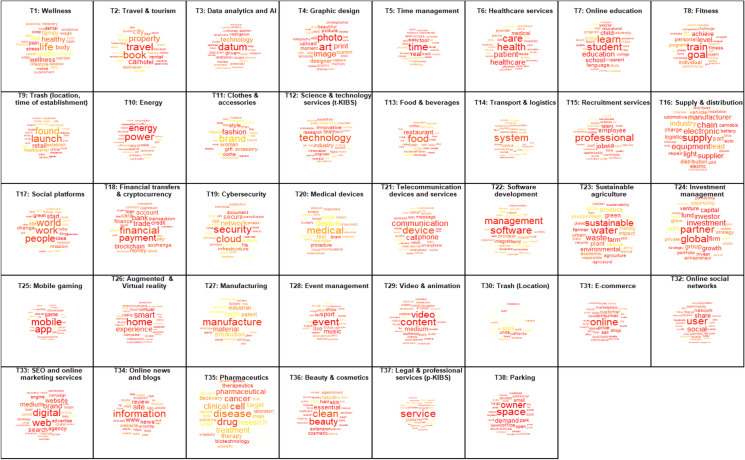
Word clouds of 38 topics generated based on full descriptions of startup companies. Note: The font size corresponds to the probability (weight) of the respective word given the topic, while the color of the word corresponds to its exclusivity (the darker the color, the more exclusive are the words)

Table 1 offers a description of the 38 identified topics based on the full descriptions of 250,226 startups in our sample. It shows the most discriminating (frequent and exclusive) words by topics and the share of the text belonging to each of the topics (topic prevalence), whereas Table 4 in Appendix 2 gives one illustrative statement per topic.^5^ After exploring the most frequent and exclusive words for each topic (see Table 1 and word clouds in Fig. 3) together with their illustrative responses, we come up with topic labels that reflect the main classes the startups belong to in a clear and concise way.

We have identified two topics that cluster information based on location and the year of establishment of the startups without any clear link to the business focus of the company. For instance, startup “Help on the way” is only described as “A program initiated and managed by Dor LeDor. It began operation on June 1, 2018, with its headquarters in Tel Aviv in Israel.” For that reason, we labeled these two topics as “trash topics” (T9 and T30).^6^ Few topic labels include acronyms that are worth to introduce below. T3 is about data analytics and artificial intelligence (*AI*). T12 and T37 cover knowledge-intensive business services (KIBS). While T12 is about startups with high use of scientific and technological knowledge (such as R&D services and engineering), i.e., *t-KIBS*, T37 focuses on traditional professional services—legal, accountancy, management, and marketing services (*p-KIBS*). T33 in turn covers services of improving the quality and quantity of website traffic to a website or a web page from search engines, i.e., search engine optimization (*SEO*). Based on these 38 topics, we offer a new way to non-exclusively classify the startups from the Crunchbase database.

Note: The terms shown are those that are the most frequent as well as exclusive for each topic. Labels for each topic reflect the content of the terms and associated startup description.

### Comparison with the Crunchbase classification

Before comparing the STM and Crunchbase classifications, one can mention that the Crunchbase classification exhibits several flaws:

1. A total of 3.2% of all startups in our dataset have missing assignment to industry classes which could bias the results on industry trends in this field.
2. Startup classifications are subject to expert bias from the side of startup founders who attribute their companies to the Crunchbase classification.
  - Industry category selection is not precise in many cases. For instance, company “Interstellar Inc” is assigned to “Information Technology” Crunchbase industry group. But, based on its full description “Interstellar is a fast secure crypto wallet and decentralized exchange, powered by the Stellar network. Our Mission is to be a true Decentralized Wallet and Exchange that allows cryptocurrency users to securely and instantly trade and transact in a peer to peer manner,” one can conclude that it is more about financial operations, transfers, and blockchain.
  - Some companies reflect redundant classes in their profiles. This is because Crunchbase recommends startup founders to choose 2–5 categories but introduces no strict limit. Moreover, it encourages to select more categories to increase visibility of the company in the database by adding less relevant classes. For instance, startup “Gooroo” with the description “On a mission to reimagine education, unlock every student’s potential, and promote lifelong learning for all. Gooroo is a monthly subscription learning platform that matches you to the perfect tutor, the best learning programs, and the most helpful educational guidance—all powered by AI. Gooroo assesses needs, pinpoints learning styles, and continuously tracks progress with data-driven insights from personalized learning experts. We’re also transparent and fair about our pricing, which is surprisingly new in education. Gooroo is where you go to get an honest education.” is assigned to an extensively large number of categories including Apps, AI, Data and Analytics, Education, Information Technology, Internet Services, Messaging and Telecommunications, Mobile, Science and Engineering, and Software, whereas Messaging and Telecommunications and Science and Engineering seem to be redundant in the list.
  - Some companies, in contrast, reflect an insufficient number of classes in their profiles. For instance, the startup “Economiza Club” with the description “Economiza Club is a collaborative platform that allows consumers to have access to actual prices in the stores in your area” is assigned only to the category “Software,” whereas “Commerce and Shopping” or “Consumer goods” are potentially missing.
3. Crunchbase industry classes are often too narrow or too wide ranging from less than 1% for Government and Military to almost 38% for Software of all startups with the mean being 6.4%, standard deviation 6.8%, and the coefficient of variation being 1.06. This makes the classes hardly comparable. Software, for example, could be related to (cyber)security, data analysis, games, and many other areas.

For comparison, the STM (topic-based) classification we provide:

1. Attributes all startups to one or more topic classes (see Fig. 5 in the next subsection) without exception;
2. Is free from expert bias making objective classification based on the startup description provided;
3. Results in topic proportions distributed more evenly with mean being 2.6%, standard deviation 1.4%, and the coefficient of variation being 0.52.

**Fig. 4:**
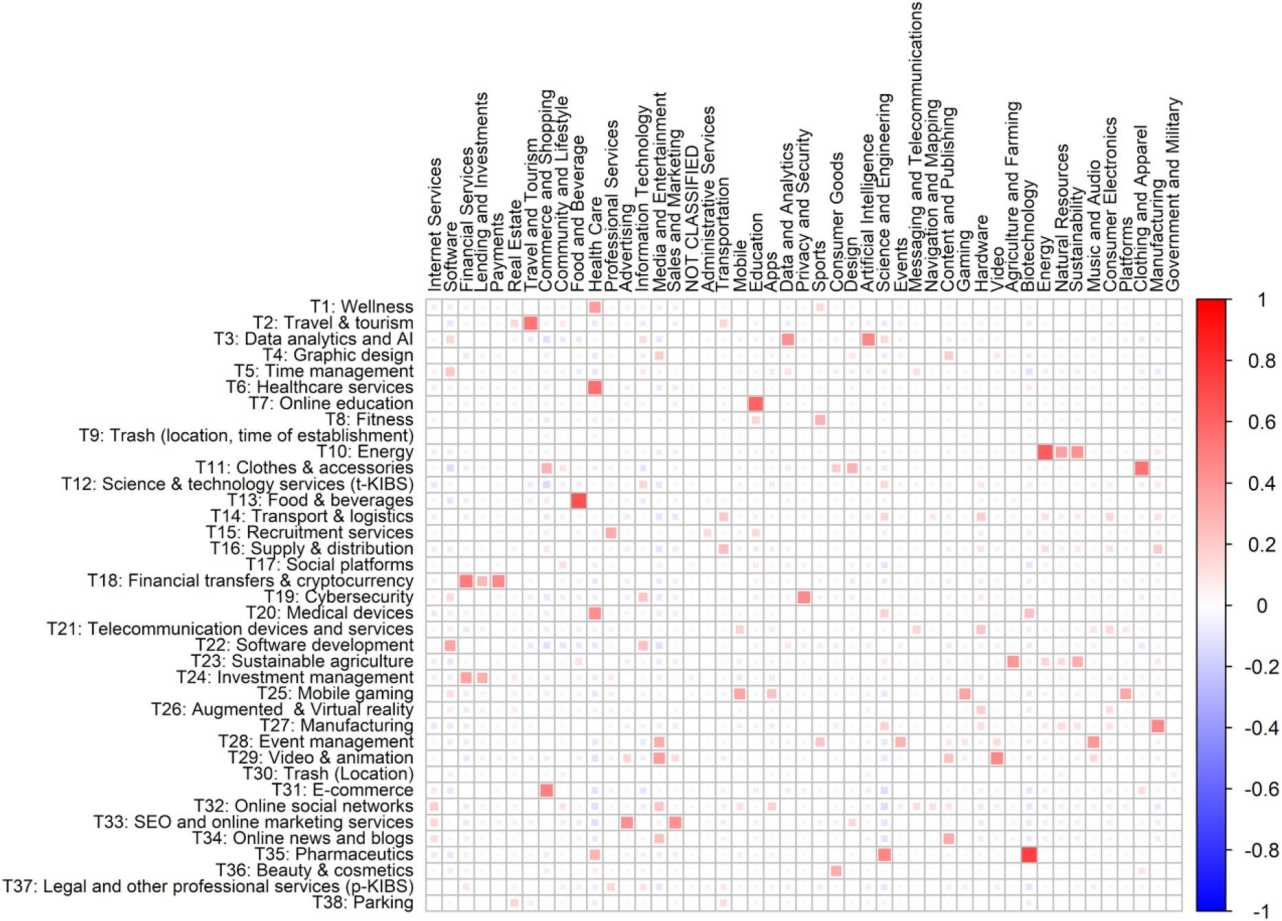
Topic co-occurrence between the STM and Crunchbase startup classifications

Finally, we examine topic co-occurrence between the two startup classifications. In particular, we measure covariance between topic prevalences for the two classifications (Fig. 4).

In comparison to the Crunchbase classification, STM approach produces topics that are better distinguishable and more concrete. For instance, the vast Crunchbase group “Software” in our classification is split into “Data analytics and AI,” “Time management,” “Software development,” and “Cybersecurity.” Similarly, the extensive “Health Care” Crunchbase group is converted in our classification to several classes: “Wellness,” “Healthcare services,” “Medical devices,” and “Pharmaceutics.” Reversely, several classes in the Crunchbase database around financial services (“Financial Services,” “Lending and Investments,” “Payments”) are united in the new classification under a single topic “Financial transfers & Cryptocurrency.” STM classification excludes too narrow Crunchbase topics, which are not widely represented in the database, such as, “Government and military” or “Navigation and Mapping.”

### Startup multidisciplinarity in the STM classification

**Fig. 5:**
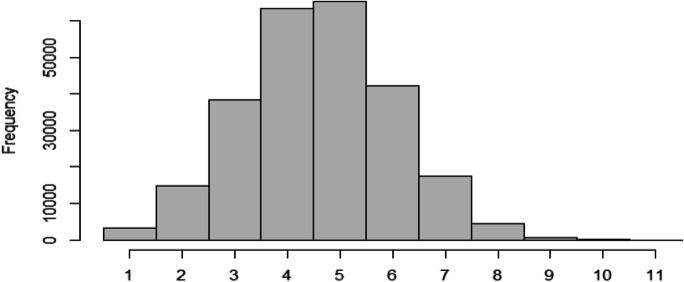
Distribution of the number of topics per startup

As the classification we propose is non-exclusive, allowing the same textual descriptions to contain fragments belonging to multiple topics, we analyze how many topics on average each startup belongs to, how this number is changing over time among established startups, and which topics tend to be recombined more (less) often. To answer the first question and accounting for the fact that the topic prevalences never equal exactly zero, we introduce a threshold of 5% that a topic should surpass to be considered as present in the description.^7^ This way we exclude the chance that words belonging with a small probability to a different topic can be misinterpreted as a signal of another class the startup description belongs to. The resulting distribution of descriptions is presented in Fig. 5. As one can see, most of the descriptions have 2–7 topics, with the mean (median) being 4.6 (5). We observe, thus, that the vast majority of the startups recombine several topics illustrating their multidisciplinary nature.

**Fig. 6:**
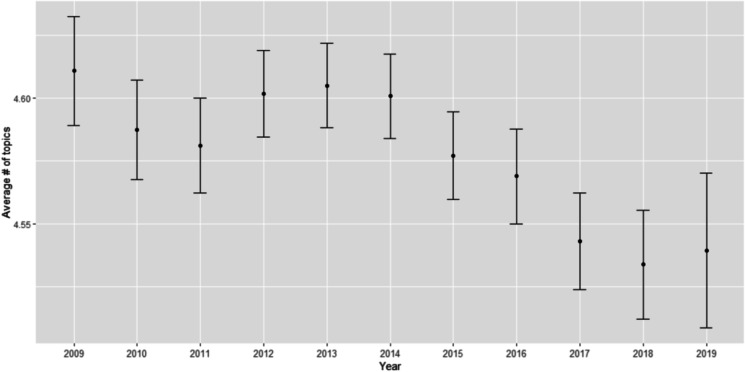
Average number of topics per startup over time. Note: Error bars represent + / − 2 standard errors

Furthermore, we plot the average number of topics per startup description over time (Fig. 6). While the number of topics per startups remains relatively stable (within the range 4.5–4.7 topics per startup), we find a statistically significant trend of slightly reducing multidisciplinarity of topics.

**Fig. 7:**
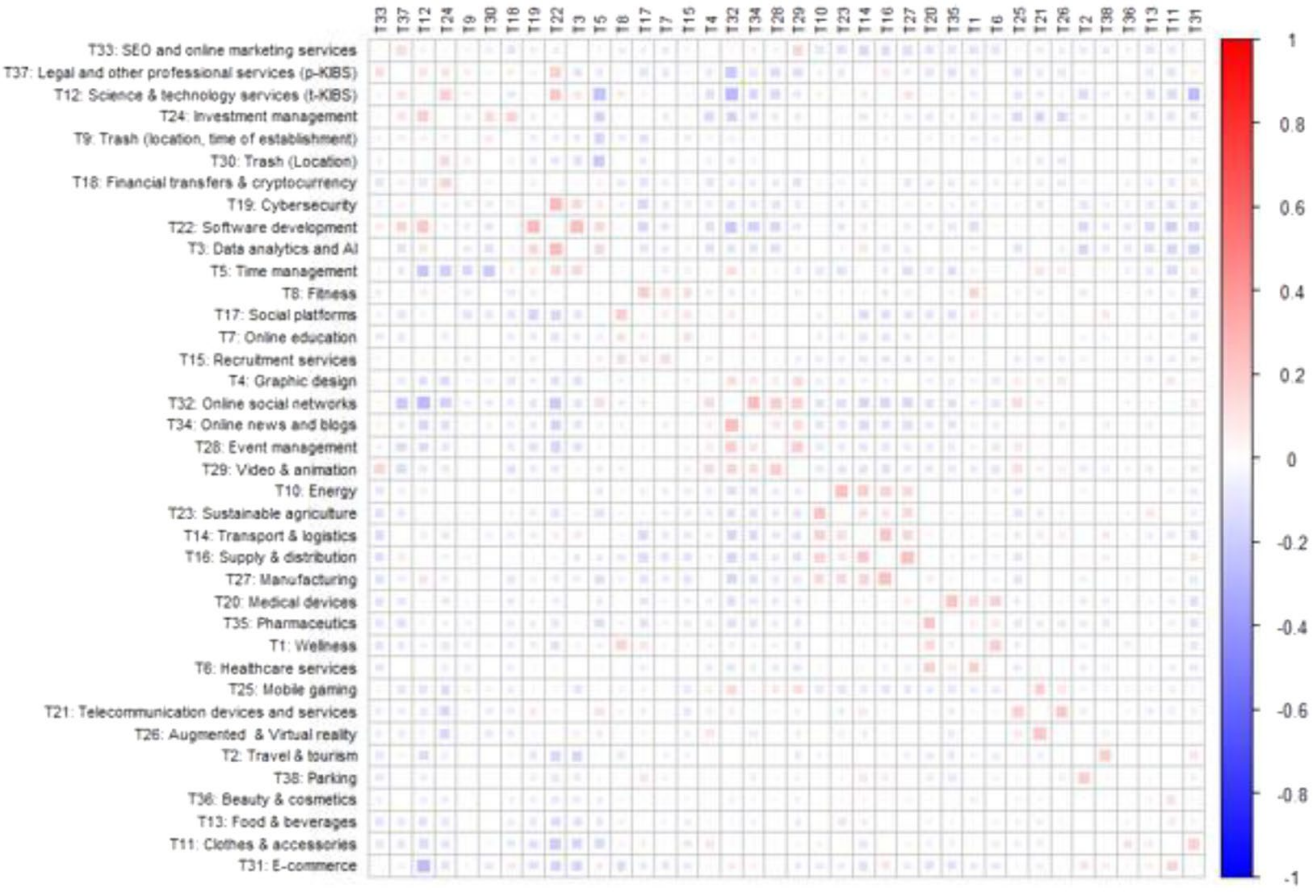
Topic co-occurrence in the STM classification. Note: The order of topics results from hierarchical clustering which positions more correlated topics closer together

Finally, let us look at what topics tend to more frequently co-occur within the startup descriptions. To this end, we plot the covariance matrix of topic prevalences. Here we do not use any threshold on topic prevalence considering also values less than 5% (Fig. 7).

We find some topics strongly overlapping in the same startup descriptions. These combinations show that certain areas tend to be combined by startups into new businesses. These are usually complementary services, which often emerged and became popular thanks to widespread diffusion of ICT technologies:

1. SEO and online marketing services (T33) and legal and other professional services (T37). Many companies such a Tokyo-based BIJIN & Co offer services related to business sales, advertising, publicity, sales promotion, and marketing research. Since clients are typically interested not just in advertising but comprehensive market research and sales strategy, companies offering these services naturally expand their specialization.
2. Investment management (T24), financial transfers and cryptocurrency (T18), and science and technology services (T12). As Saiedi et al. (2021) recently pointed out, many new startups active in the financial sector—such as New York–based Templum—start using blockchain technologies to offer transparent and secure financial investment management services for their clients.
3. Software development (T22), data analytics and AI (T3), science and technology services (T12), cybersecurity (T19), and time management (T5). This cluster represents a combination of activities with similar (computer science) skills required: developing software, providing cybersecurity, and data analytics. One example of such startup is Tel Aviv–based Vulcan Cyber. As its description states it is “security company that helps enterprises quickly detect and fix vulnerabilities in their software stack and code by utilizing Cloud technologies, scanning tools and Big Data as well as complex decision systems.”
4. Fitness (T8), social platforms (T17), online education (T7), and recruitment services (T15). As outlined in the description of Story2, the startup employs “online group coaching […] teaching unemployed, under-employed, and unhappily employed millennials to connect their skills, experience, and temperament with high-impact work.” Such companies provide online education and training for large masses of people making knowledge more accessible than it was ever before. Allen et al. (2016) confirm presence of the online education platform trend, with the share of students enrolled in the distance learning courses as well as spendings of institutions on this type of education growing.
5. Online social networks (T32), graphic design (T4), online news and blogs (T34), event management (T28), and video and animation (T29). This cluster demonstrates addition of content services to social networks in the form of news, discussion groups, or video sharing. One of the projects in this field is SQUID. It offers an app for curating, reading, sharing, and discussing news on different topics that can be specified by the user.
6. Topics related to production: manufacturing (T27), transport and logistics (T14), supply and distribution (T16), energy (T10), and sustainable agriculture (T23). Startups in these classes offer services along the entire value chain. In line with Wang and Hsu (2021), startups in smart manufacturing integrate “production, warehouses, logistics, and even environmental and social requirements to create the digitization of the automated manufacturing environment.” They establish global value creation networks with efficiency and productivity improvements among firms across the whole value chains. For example, Sense Photonics is one such company. They are specializing in LiDAR and 3D sensor technologies that can be employed in production, storage, and transportation. Description of the company highlights that the solution is robust, cost-, and energy-efficient.
7. Healthcare services (T6), wellness (T1), medical devices (T20), and pharmaceutics (T35). This combination is largely due to emergence of many mobile health applications offering wellness management, self-diagnosis, medication reminder, and other services. These startups aim to provide low-cost, around-the-clock access to health information for their clients. As Kao and Liebovitz (2017) point out, however, these startups face multiple barriers, including lack of regulatory oversight, limited evidence-based literature, and concerns of privacy and security.
8. Mobile gaming (T25), telecommunication devices and services (T21), and augmented and virtual reality (T26). With the growing power of computers and progress in AR/VR technologies, many startups—including LyteShot—aim to create unique gaming experiences users can have with their PCs and smartphones. This trend has been recently discussed by Liao (2019) pointing that AR is an important area that will shape evolution of mobile technologies.
9. Travel and tourism (T2) and parking (T38). Sharing economy provides examples of several models in transportation sector which transform the way people travel. One of the examples is provided by Jian et al. (2020) who propose a novel solution of parking, integrating carsharing platform with parking sharing. Our dataset contains several companies that facilitate sharing of the parking spaces. Kerb in Australia, ParkBee in Europe, NOSON in the USA. They all offer a convenient way to share your parking lot during the time you are not using it. It greatly simplifies traveling and parking.
10. E-commerce (T31) and clothes and accessories (T11). Existing literature emphasizes increasing role of technological development changing the shopping behavior of customers: usage of connected devices and online retail platforms (Reinartz et al., 2019), rising novel marketplace channel between manufacturers and e-tailers (Yan, et al., 2018), and adoption of autonomous shopping systems (De Bellis & Johar, 2020). We also see that the online sales channel stimulated new developments in clothing market. For example, Neronote company allows its clients to customize their clothes by choosing material, certain elements, and type of fit they like the most while maintaining a mass-market price of the clothing item. This combination only became viable with the use of online technologies.

As one can see, some of these combinations are demand-driven, where customer needs span over a number of services provided by the startup companies (e.g., advertising and professional services or renting a car and parking it). In other cases, combination of services creates a synergy effect, e.g., in terms of lower cost or higher quality (take for example online education and healthcare services or online e-commerce). Finally, sometimes the combinations may be driven by the skills available in the startup, where same people with computer science skills can develop software, offer cybersecurity and data analytics services.

### Dynamic shifts and geographic concentration

Now we analyze the role of covariates in explaining the variation of topic prevalences among the startup descriptions. We provide results from regression analysis of topic proportions over the seven explanatory variables we used in constructing our topic model across time and location (six dummy variables were used for each of the regions in the Crunchbase classification).^8^ In particular, a linear regression model was specified for each of the 38 topics (indexed by *k*) as follows:

$${Topic Prevalence}_{k} \sim {Constant}_{k}+ Year+ Locatio{n}_{NA}+Locatio{n}_{Eur}+Locatio{n}_{Asia} + {Locatio{n}_{SA}+Locatio{n}_{Oceania}+Locatio{n}_{Afr}+Residual}_{k}$$

**Fig. 8:**
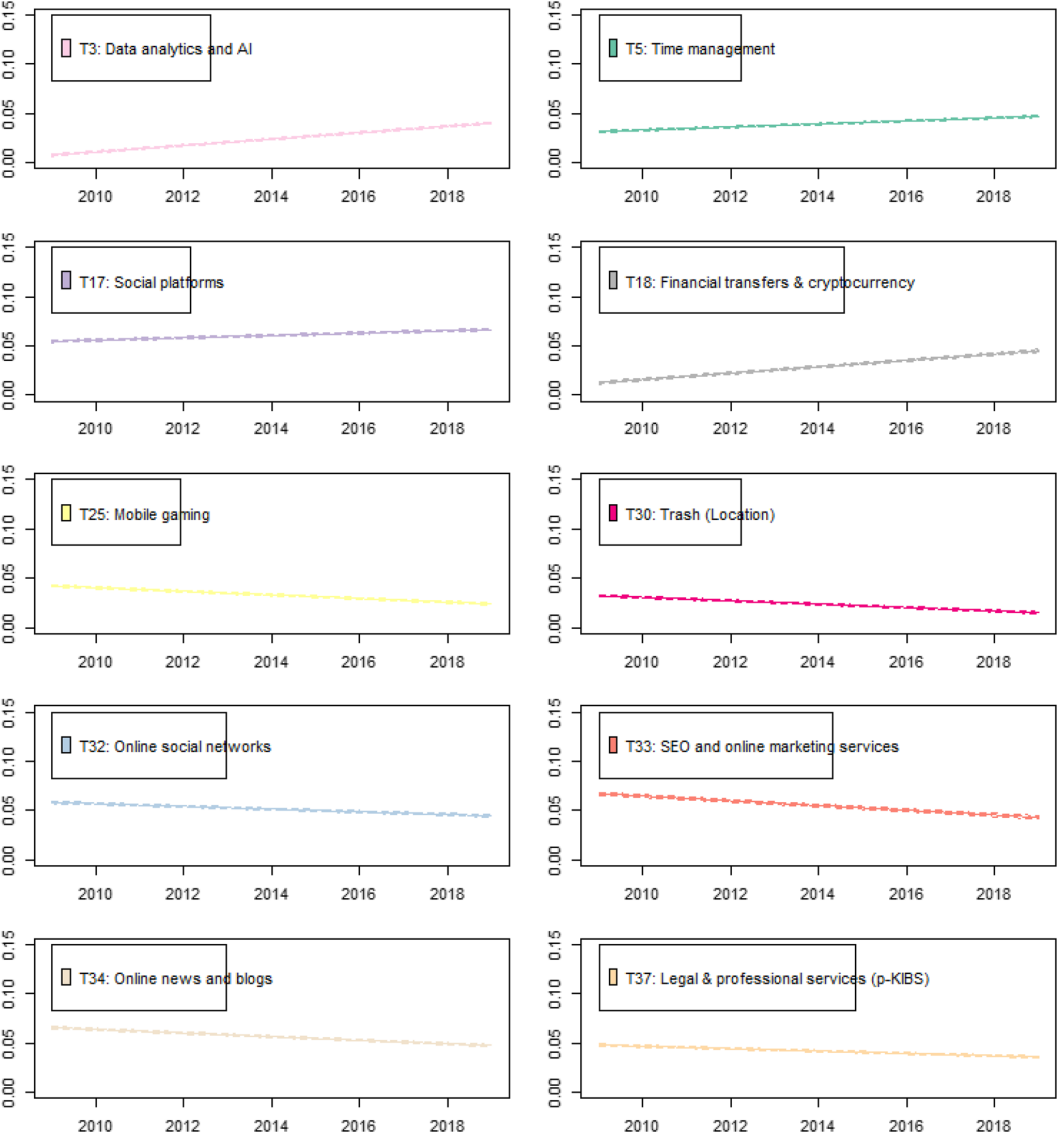
Time trends of startup activity. Note: Only results with a regression coefficient in absolute value above 0.001 and significance at least 0.1% are reported

Figure 8 presents the resulting time trends with the steepest (regression coefficient in absolute value above 0.001) and significant (at least 0.1% significance level) coefficients.^9^ From the graphs below, the positive time trends are observed for data analytics and AI (T3), time management (T5), social platforms (T17), financial transfers, and cryptocurrency (T18). These findings are supported by the indirect data sources and several analytical reports. For instance, according to Google Trends, interest in the crypto economy has been growing in recent years: the number of searchers for the word “bitcoin” has increased three-fold, and for the word “cryptocurrency,” eight-fold for the period May 2020–May 2021. Bitcoin price is also steadily growing, and it is now six times higher than a year ago. CB Insights reports display that since 2014, more than 4.3 thousand AI startups have raised equity funding globally; venture capital investment in this field hit 26.6 billion USD worldwide in 2019, which is six times higher than in 2014 (CB Insights, 2020).

The negative trends are observed in mobile gaming (T25), online social networks (T32), SEO and marketing services (T33), online news and blogs (T34), and legal and professional services (T37). As reported by CB Insights (2017), the digital media startup landscape (podcasts, news websites, blog syndicates, newsletters, video sites, and any other content apps and sites, excluding user generated content and social networks) is on the decline. In contrast to our findings, recent reports claim that mobile gaming is on the rise. For instance, Newzoo, a provider of gaming and e-sports analytics, shows that the gaming market (including PC, console, and mobile gaming) will continue to grow in the following years, exceeding 200 billion USD at the end of 2023 (Newzoo, 2020). This seeming contradiction could be explained by the consolidation of the market, fast growth of incumbent firms, and high entry barriers for startup companies. More research is needed to answer this question.

**Fig. 9:**
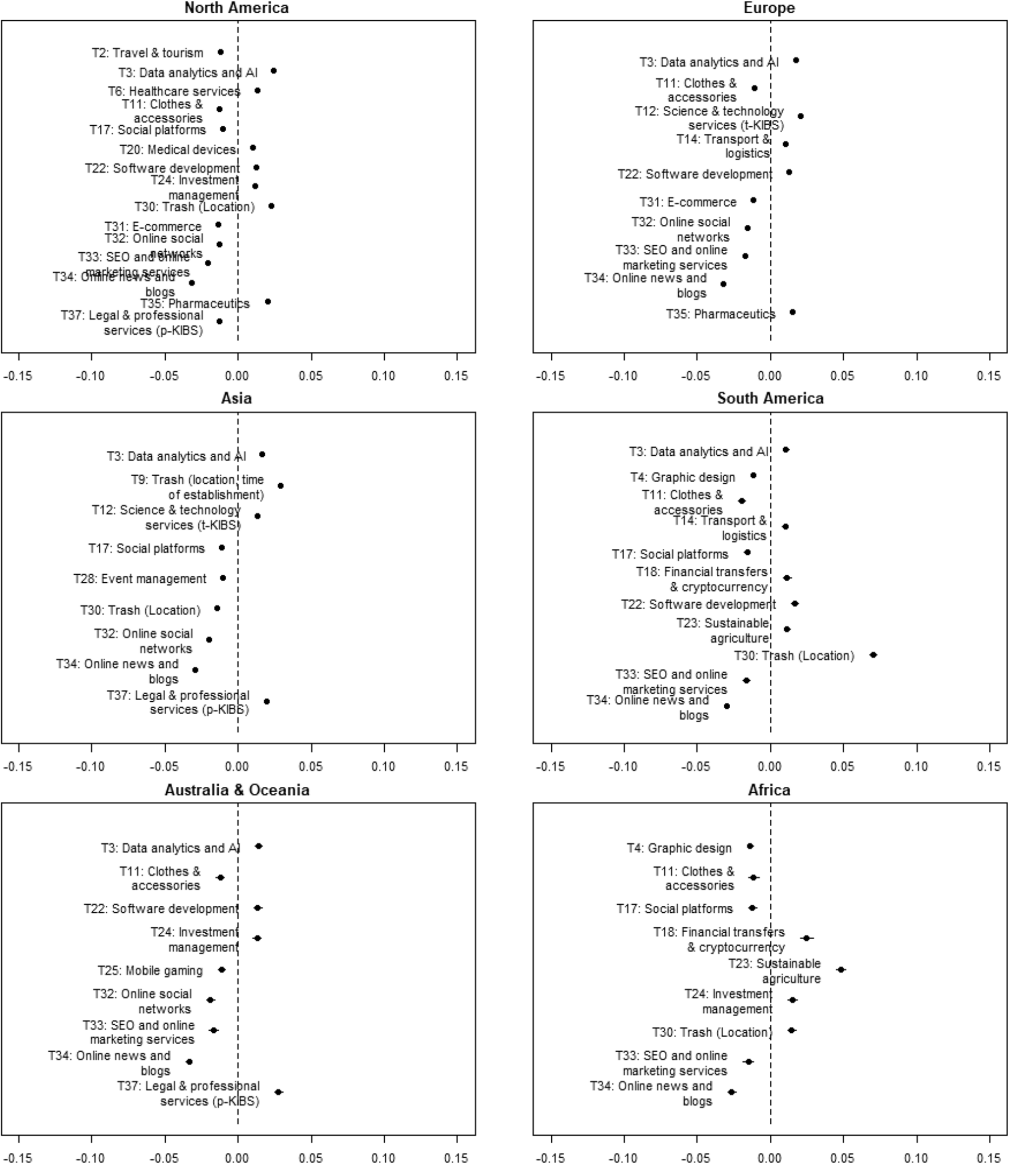
Geographical concentration of startup activity. Note: Only results with a regression coefficient in absolute value above 0.01 and significance at least 0.1% are reported. The plots show mean difference in topic proportions between the region under consideration and the rest of the world (a positive value on the X-axis indicates a larger prevalence in the corresponding region)

Graphical representation of geographical concentration is provided on Fig. 9. Similarly, only results with a regression coefficient in absolute value above 0.01 and a significance level of at least 0.1% are presented in the figure, while all the results are reported in Appendix 2. Figure 9 shows that prevailing startup industries differ depending on the headquarter location of startups.

Startups located in North America tend to develop in the fields related to information technology (T3: data analytics and AI; T22: software development), medicine (T6: healthcare services, T20: medical devices, T35: pharmaceutics), and finance (T24: investment management). These findings are supported by analytical results from available market reports in the field of technology entrepreneurship. For example, European AI Landscape Research affirms that the USA is the global market leader in AI with a 40% market share (Berger, 2018).

Startup industries dominating in the European market are similar to North America: startup projects tend to be launched in high technology and IT sectors (T3: Data Analytics and AI; T22: Software development; T12: Science and technology services t-KIBS), pharmaceutics (T35), and transport and logistics (T14). Pharmaceutical market tends to grow in both North America and Europe, which is confirmed in the iQvia Institute report (2019): the largest market shares refer to the US market forecasted at 625–665 billion USD by 2023 (growth of 4–7%), and European market at $195–225 billion (growth of 1–4%).

Analysis by the McKinsey Global Institute (2019) infers that Asian market comprises more than 30% of the global “unicorn” companies (startups valued at more than one billion USD). One of the key success factors is proved to be the rich venture capital market of the region. Asia is now one of the leading global venture capital markets in virtual reality, autonomous vehicles, 3D printing, robotics, drones, and AI. These facts coincide with the findings of our analysis: Asian founders tend to generate proportionally more startups in science and tech services (T12); data analytics and AI (T3); and legal and professional services (T37).

Considering the African technology market, startups tend to arise in the fintech (T18: Financial transfers and cryptocurrency; T24: Investment management) and agritech sectors (T23: Sustainable agriculture) which is confirmed by media reports in Business Insider Africa (Oluwole, 2020), Partech Partners (2020), Briter Bridges (2021), and Disrupt Africa (2020). Startups located in South America are concentrated in the fields of software development (T22), transport and logistics (T14), financial transfers and cryptocurrency (T18), and sustainable agriculture (T23). Startups headquartered in Australia and Oceania tend to focus more on investment management (T24), software development (T22), and legal and professional services (T37).

### Distribution of investments across startup classes

Finally, let us consider the distribution of venture capital investments among startup companies in our sample. For this, we collect data on the funding raised by the companies in our sample. It is proxied by the indicator of total funding amount that represents cumulative investment raised by the considered startup across all funding rounds (angel, pre-seed, seed, etc.). Investment raised by the startups in our sample is 1.4 trillion USD in total. Unfortunately, this data has a lot of missing observations: out of 250,252 companies in our sample, only 71,910 startups have information on the total funding amount. One could explain so many missings by the nascent nature of firms registered in Crunchbase: many of these companies are registered in Crunchbase before they receive funding (Yu, 2021). Nevertheless, it is worthwhile to see how shares of our startup classes change, if we weight them by the amount of funding they received.

**Fig. 10:**
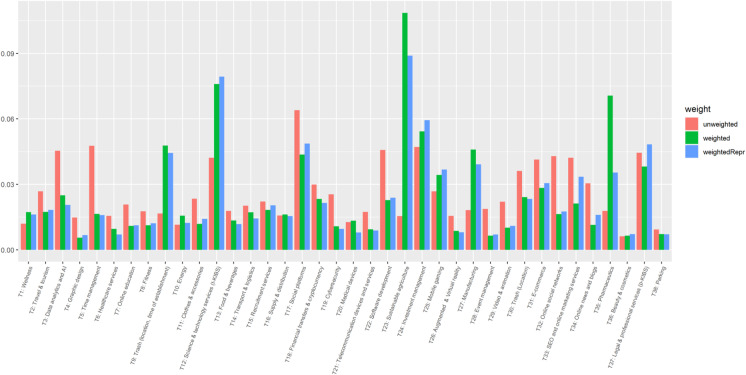
Shares of classes from the STM classification before and after accounting for the amount of investments received. Note: “unweighted” represents the weights obtained by summing up the shares of classes across the full sample of 250 thousand startups not taking investments into account; “weighted,” weights obtained by multiplying the prevalences of the smaller sample of 72 thousand startups by the amount of investments received by each of this startup; “*weightedRepr*,” by multiplying the “weighted” shares with the ratio of the number of startups in our overall sample belonging to this class and the number of startups belonging to this class for which the amount of investments is known

**Fig. 11:**
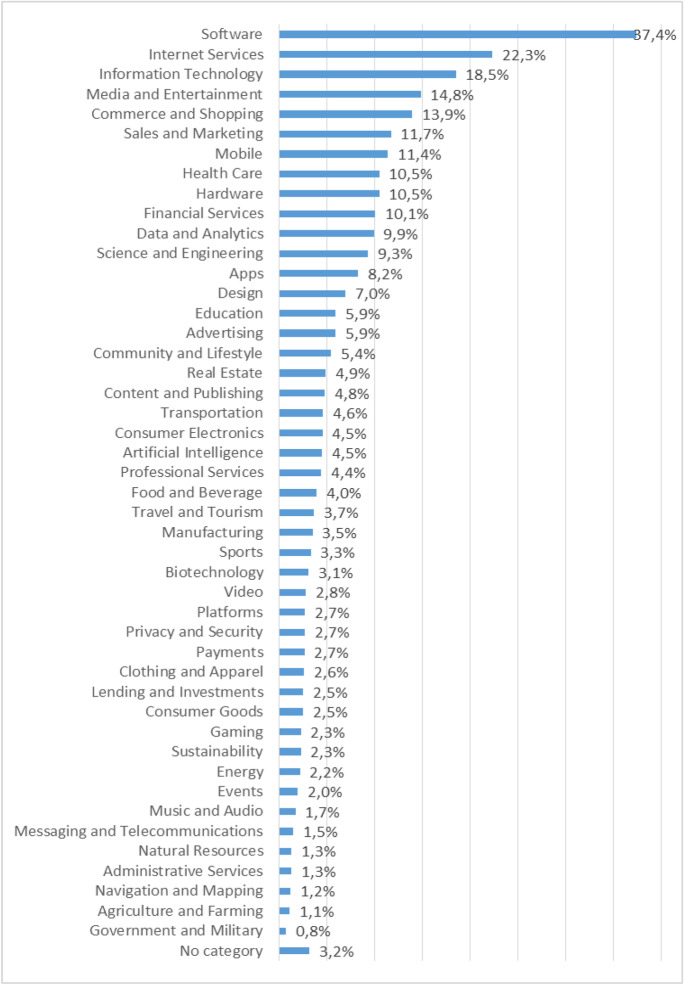
Shares of classes in the Crunchbase classification for the period 2009–2019 based on the same sample of 250,226 startups

Figure 10 reports shares of the 38 classes in three forms: (1) the weights obtained by summing up the shares of classes across the full sample of 250 thousand startups not taking investments into account, i.e., the topic prevalences reported in Table 1 (in the following “unweighted”); (2) the weights obtained by multiplying the prevalences of the smaller sample of 72 thousand startups by the amount of investments received by each of this startup (in the following “weighted”). Note that this version essentially disregards startups for which the amount of investments is unknown; (3) since the information on investments is available not necessarily randomly and to correct for the resulting bias, we introduce a third option where we multiply the “weighted” shares by the ratio of the number startups in our overall sample belonging to this class and the number of startups belonging to this class for which the amount of investments is known. This way we can account for the fact that the number of observations on investments is not necessarily randomly distributed. However, here we still assume that the observations available for each class are representative, which does not have to be the case. This last option is depicted in the following as “weightedRepr.”

As one can see, after taking the amount of investments received into account, we find a few classes such as Sustainable agriculture (T23), Science and technology services (T12), and Pharmaceutics (T36) considerably increasing their share in the overall classification, while classes like Data analytics and AI (T3), Time management (T5), Cybersecurity (T19), and Event management (T28) shrinking. One could explain this by higher requirements startups in the former classes should satisfy (e.g., having unique technology, laboratory, and equipment) compared to startups in the latter classes. This can cause lower competition at the stage the startups are founded in the former classes, stimulate investors to provide venture capital, and require larger investment to proceed with scaling up the production. Another reason is the unprecedented penetration of new technologies (AI, cloud computing, gene editing tools) in agricultural and health sectors (Savin et al., 2022), which attract new investors. An additional reason for increasing attractiveness of startups in pharmaceutics is the shift of biopharmaceutical drug development from in-house production of large pharma companies to small and mid-sized companies to diversify risky internal R&D programs (Melchner von Dydiowa et al., 2021; Murphey, 2019). With the rapid advances of new technologies in this sector, large pharma companies appear as investors for initial drug development that boosted funding of biopharmaceutical startups over the past decade.

## Conclusions

This study provides analysis of global startup activities based on their textual descriptions in the Crunchbase database, one of the leading platforms aggregating data on startups. Our data comprises information on 250,252 textual descriptions of startups founded in the period of 2009–2019. Using topic modeling, we provide a novel classification of startup companies free from expert bias containing 38 topics. By taking into account the year of establishment and the geographical location of the startups, we measure which topics were increasing or decreasing over time and which of them were concentrated in Europe, North America, or other regions. We compare our classification to the one existing in the Crunchbase database demonstrating how we improve upon it by relaxing its limitations. In particular, 3.2% of all startups are not attributed to any class; attribution of startups by their founders to the existing classes is prone to expert bias, where companies sometimes refer only to one category instead of many and vice versa; Crunchbase industry classes are often too narrow or too wide (ranging from 1 to 38% of all startups). In comparison, our approach classifies all startups without exception, and produces classes that are better distinguishable and more concrete. The resulting topic-based classification exhibits more even distribution of class shares.

Statistical analysis of topic proportions across time and regions allows us to report significant trends and regional differences among the identified startup classes. Upward trends are detected in the fields of data analytics and AI, time management, social platforms, and financial transfers and cryptocurrency. Downward trends are found in mobile gaming, online social networks, SEO and marketing services, online news and blogs, and legal and professional services. We also find that startups located in North America tend to appear in the fields related to information technology (particularly, data analytics, software development), medicine (particularly, healthcare services, medical devices, and pharmaceutics), and finance (particularly, investment management). European startup projects tend to be launched in high technology and IT-sectors (particularly, data analytics and AI; software development; science and technology services t-KIBS), pharmaceutics, and transport and logistics. Asia hosts a large fraction of startup companies in the science and tech services, data analytics, and legal and professional services.

Furthermore, we find groups of classes which startup companies tend to combine to provide novel products and services. Some of these combinations are demand-driven (e.g., social network combined with news or renting a car and parking it); others create a synergy effect in terms of lower cost or higher quality (e.g., online education and healthcare services, online e-commerce). This information can be instructive for existing entrepreneurs on how they could diversify their businesses. Also, it can be useful to determine actors competing for the same customers since industry classification is very imprecise for this purpose (Cantner et al., 2019; Savin et al., 2019).

Finally, we analyze how the shares of startup classes change after we take the amount of investments they collected into account. As a result, classes on sustainable agriculture and pharmaceutics considerably increase their share at the cost of classes related to data analytics and time management. This may be due to higher requirements startups in the former classes must satisfy (e.g., having unique technology, laboratory, and equipment) and as a result, lower competition for investments.

Our findings can be of interest to startup founders, investors, and policy makers, as they elicit the hidden topic-based classification of startup companies around the world and dominant trends for these companies. Founders and startup executives could consider these results as useful insights for decision-making, where to establish the headquarters or additional offices to maximize knowledge spillovers and become profitable, and which industry is currently on the rise and is potentially more attractive for funding. Venture capital investors could benefit from the information on the geographical distribution of companies to anticipate future market leaders and unicorn companies. Policy makers could rely on our findings for development of local policies to support startup ecosystems in targeted industries. The fast evolving or, contrary, lagging industries require specific policy regulations based on country development and current technology trends. For example, existing literature provides evidence that fintech, one of the fastest growing industries nowadays, started to compete with traditional financial institutions and increased the complexity of competition policy in the USA (Van Loo, 2018).

Our study has many directions for future research. As we described in our analysis, the information on the amount of investments received has too many missing observations in the Crunchbase database. To address this question, one could combine Crunchbase with other established sources collecting information on startup activity, i.e., PitchBook, LinkedIn, AngelList, and Startup Ranking. This could provide further insights on financial performance of startups and differences between startup classes. A similar comment applies to the operating status of startups in the Crunchbase database. While this data is available, checking the status of a small sample of companies by searching for their website showed that many “active” companies are not active anymore. Hence, before using this information, one should ideally double check the status of the companies in the other sources. Furthermore, one could collect data that has been added to Crunchbase over the last year and measure how shares of the described topics have changes, or whether new topics (for example related to COVID) have appeared.

As for limitations, our study is dependent on availability and the quality of the full startup descriptions provided by their founders. These descriptions are vital for completeness and accuracy of our textual analysis. If no full description is provided, we cannot cover that startup in our analysis, which biases our sample. The more detailed and precise are the descriptions, the better are our results. Therefore, a way to improve the results is to collect further information describing each startup from external online sources including those listed above. Another limitation is the country coverage in Crunchbase. While North America, Europe, and Asia are well-presented in the database, this platform is less known in South America and Africa. Therefore, our findings on these regions must be taken with a grain of salt.

## Data Availability

The data used can be provided on request.

## Appendix 1 Crunchbase classification

**Table 2:** Geographic distribution of startups before and after cleaning, in percent

Headquarter location	Before cleaning	After cleaning
Africa	1.4%	1.4%
Asia	16.2%	16.3%
Europe	24.8%	24.7%
North America	40.1%	40.5%
Oceania	1.9%	1.9%
South America	3.0%	3.0%
Location undefined	12.6%	12.1%

**Table 3:** Distribution of startups before and after cleaning, by year of establishment, in percent

Year of establishment	Before cleaning	After cleaning
2009	7.3%	7.3%
2010	8.7%	8.7%
2011	9.5%	9.5%
2012	11.2%	11.2%
2013	11.6%	11.7%
2014	11.4%	11.5%
2015	11.1%	11.1%
2016	9.5%	9.5%
2017	9.0%	9.0%
2018	7.1%	7.1%
2019	3.6%	3.5%

## Appendix 2 Further results on topic-based startup classification

**Table 4:** Illustrative responses for the identified topics

**Topic 1. Wellness** Anybody coping with anxiety, anger, substance abuse, depression, or relationship problems can benefit from some therapy. That is why Baton Rouge Counseling offers high quality therapists who are ready to help you make a positive change in your life
**Topic 2. Travel and tourism** CheckYeti is the largest online platform to book activities in 1000 + mountain and beach destinations across Europe—including skiing and snowboarding, rafting and canyoning, mountain biking, paragliding and ballooning, diving and surfing, jet skiing, boat trips, and many more. With 12,000 + offers in 16 countries, CheckYeti enables holidaymakers and outdoor enthusiasts to experience summer and winter activities with their ideal guide
**Topic 3. Data analytics and AI** NeoWize changes the way we look at machine learning and deep learning. Current deep learning algorithms focus on making the most out of the data available. NeoWize utilizes neural networks and adaptive input to create more data and better data, thus increasing our predictive power with small datasets
**Topic 4. Graphic design** Ahsania e Solutions is a graphics design company and our services include in design page format, clipping path, image masking, edit picture, image editing, and neck joint
**Topic 5. Time management** Online calendar and scheduling tools to make scheduling meetings simple and efficient. The inefficiency of scheduling meetings is costing your business. Introducing Skedgit, a new way to coordinate meetings!
**Topic 6. Healthcare services** Revon is building a platform to connect physicians and patients. The Revon Platform will allow physicians to deliver care to patients remotely, and allow patients to manage their health better
**Topic 7. Online education** Beaver Math is a professional online education company. Beaver provides high-quality online mathematics courses for primary and secondary school students and schools
**Topic 8. Fitness** AthleteProgress systematically guides athletes and coaches through purposeful, focused, and deliberate practice. AthleteProgress quickly defines strengths and weaknesses, helps to develop long-lasting habits, elevates athletes to higher levels of performance, and guides rapid goal setting and achievement
**Topic 9. Trash (location, time of establishment)** Sensus operates as a stealth mode company. The company was founded in 2018 and is headquartered in Tel Aviv, Israel
**Topic 10. Energy** SolVera Energy is a renewable energy development company providing clean energy development and advisory services to the commercial, industrial, and utility marketplace. SolVera Energy is engaged in the development, financing, ownership, and operation of renewable energy projects. Our projects include distributed and utility scale photovoltaics, wind, energy storage, cogeneration, and biomass fuels and power generation
**Topic 11. Clothes and accessories** Ethnic Fashion store for traditional Indian wear. Our one stop online platform offers the widest variety of ready to ship, custom stitch, popular, and trendy ethnic fashion; our online store offers it all from bridal/wedding sarees and lehengas to contemporary Indo-western outfits, traditional wedding accessories, Salwar, anarkali dresses, kurtis, designer collection, party wear Bollywood look saris, and more
**Topic 12. Science and technology services (t-KIBS)** The Netherlands eScience Center is the Dutch national center of excellence for the development and application of research software to advance academic research. They are convinced that research in every academic discipline can be improved by taking advantage of available digital technology. They take a multidisciplinary approach, combining their deep knowledge of both academic research and software development to help define and solve research challenges
**Topic 13. Food and beverages** Simply Prep Meals specialize in premium quality meal plans and build-your-own meal prep. They cook and freshly prepare healthy ready to eat meals and delivery them fresh, cool, and always free across the UK. Prep made simple
**Topic 14. Transport and logistics** Astral Aerial Solutions is a UAV operator and service provider. It offers drone services that provide a new dimension to logistics, aerial photography, aerial surveillance reconnaissance, and inspections among other industries. Its cargo drone has a payload capability of up to 2000 kg, 1200-km range, and flight time of up to 26 h on surveillance mode. A smaller drone is capable of 8 h of flight carrying up to 4 kg payload. These drones are suitable for humanitarian cargo air transport, medical deliveries, aerial mapping and imagery, aerial surveillance and security, agriculture, oil and gas services, connectivity, and emergency response
**Topic 15. Recruitment services** WorkSumo is employment on-demand. WorkSumo’s platform connects job seekers to hundreds of job opportunities and enables employers to fill shortages and hire pre-screened reliable candidates on-demand
**Topic 16. Supply and distribution** A leading distributor for Exide batteries in Bangalore and universal batteries stocks a large supply of batteries for distribution around Bangalore. A total 2 wheeler batteries and 4 wheeler batteries are also distributed by us for battery dealers in Bangalore
**Topic 17. Social platforms** Everyone has ambitions, dreams, and goals. Some of us have bigger ambitions than others. Each ambition is as unique as the people who have them. And for every person who has an ambition, there are surely those who can assist or at least motivate them towards realizing their ambitions be they great and lofty or simple and modest. AmbitionBag is designed to help everyone with ambitions, including you. What is AmbitionBag all about? AmbitionBag is a social platform for you to share your life ambitions, help or motivate other people to achieve theirs, and also have other people help or motivate you to achieve yours. It is all about mutually helping one another. Why do you do this? Simple. By giving people the platform to help each other with their life and ambitions, we get help and motivation for our ambitions as well. All in all, the world will be a better place. How does it work? Refer to How it works page. Who is AmbitionBag for? AmbitionBag is for everyone and anyone who has ever had any ambition in their lives and who wants to help others achieve theirs. Basically you and me! When was AmbitionBag conceived? The idea for AmbitionBag was first conceived in February 2012. Who is behind AmbitionBag? We are a team of positive-minded, ambitious people. What are the ambitions of AmbitionBag? We want to make the world a more positive place. We want more people to realize happiness by having their ambitions fulfilled
**Topic 18. Financial transfers and cryptocurrency** CardAlpha is a financial platform which enables small businesses to receive customer payments, hold business funds securely in a digital account, and make business payments via electronic transfers or a business debit card. SMEs transacting on the CardAlpha platform can also access business loans at competitive rates based on their transaction history and credit bureau records
**Topic 19. Cybersecurity** FireLayers enables IT to proactively protect the usage of cloud applications via rule-based policies while ensuring that they maintain the required security and compliance levels. They protect cloud application usage from initial user identification to the safe retrieval of data and request fulfillment. They add a layer of proactive protection and monitoring to any cloud application identifying all users, applications, and actions
**Topic 20. Medical devices** Sotera is a medical device company focused on innovative solutions for cardiac procedures. The company’s initial product is a solution for reducing complications to the esophagus during catheter ablation treatment for atrial fibrillation. It is commercializing a novel medical device designed to protect the esophagus during catheter ablation procedures
**Topic 21. Telecommunication devices and services** Earin is the smallest, wireless earbuds available on the market. Two earbuds that together function as one Bluetooth headphone, no cables, no attachments, just magically small delivering high quality audio
**Topic 22. Software development** Candylio is a consulting and implementing software development tool and business management system company that assists clients in deploying software development tools, service desks, and other business critical solutions on operational levels. It aims to deliver a more effective, collaborative, and cost-effective software solution by creating a value. Company’s products include JIRA, Confluence, Service Desk, HipChat, Bitbucket, and any Atlassian product or plugin
**Topic 23. Sustainable agriculture** MicroGen Biotech uses Nature’s microbes to reduce toxins in crops while increasing crop yields and reducing pollutants in arable soil. Our vision is to feed the world with safe foods produced sustainably
**Topic 24. Investment management** Finprime is a boutique private equity and venture capital advisory firm, focused on early stage investments into growing companies, syndication of debt, and equity financing and strategy advising for growing companies
**Topic 25. Mobile gaming** A bit Games develops mobile applications for the Android and iOS users. They design applications that are related to games and are compatible with iPads and iPhones. Super Sheep Tap is one of the gaming applications launched by A bit Games
**Topic 26. Augmented and Virtual reality** At Sense Glove, we see virtual and augmented reality as enabler for real world applications. Our belief is that providing interactions with objects and persons equal to those in the real world is the only way to fully utilize the potential of VR and AR. That is why we are creating the Sense Glove; the Sense Glove provides the most natural interaction in virtual and augmented reality. With the Sense Glove virtual, objects are brought to live; you can feel, touch, and interact with them as if they are real
**Topic 27. Manufacturing** Boron Nitride Powders is a chemical manufacturing company that specializes in the production of sub-micron and nano-size particle ceramic powders. Boron Nitride Powders operates a plant to produce hexagonal Boron Nitride to be followed by plants to produce Boron Carbide and Silicon Carbide powders
**Topic 28. Event management** Danceter is the web platform for sports dance events. We help organizers of dance sports events to create and promote their competitions. We create unique and adaptive registration systems special for dance sports events. Our solution helps organizers of competitions manage and promote their event and rise quantity of dancers and audience
**Topic 29. Video and animation** vAnimations Studio is the brand of vServices Ltd and is one of the best UK animation studios that produce 2D animation, 3D animation, Explainer Videos, Promotional Videos, Whiteboard videos, and motion graphics, including Animated Songs and Children Stories
**Topic 30. Trash (Location)** CrowdTube.TV is an internet company based out of Calle Laguna del Marquesado N19, Nave 16 Edificio Adriana 1 Planta, Poligono Industrial La Resina (Villaverde), Madrid, Community of Madrid, Spain
**Topic 31. E-commerce** Delicious Coupons is an e-commerce website. It provide shopping ideas for online buyers; with the help of this website, you can get many types of coupons, some like coupon code, discount code, promotion code, free coupon code, online promotion code, exclusive coupons, e coupons, summer coupons, list of coupons, and winter coupons as well as types of coupons that are available at DeliciousCoupon.com
**Topic 32. Online social networks** AXYS allows two people to see what friends or interests they share. AXYS technology layers the Social Networks; a user selects to share so common friends and interests can be the focus of the conversation. Keep the conversation going by connecting through any of those social networks at a later date. Users can then access a history of the people they meet by remembering the when and where
**Topic 33. SEO and online marketing services** Lasso Gravo is the best Web design and SEO company in Coimbatore. Lasso can create all kinds of business website with attractive design. SEO and digital marketing also have in Lasso Gravo. Lasso is ready to improve your business online. We can provide organic result and payable result like SEO and digital marketing. SEO means search engine optimization. It will help your business website come top result without any pay search engine. Digital marketing is totally different from SEO. If you paid money to google. It will give top digital result or search result
**Topic 34. Online news and blogs** Welcome to article directory Eddy Articles‚ Free Articles Directory | Submit Articles. Find free articles to publish on your website or blogs. At Eddyarticles.com, there are many authors who have contributed high quality articles. There are many unique high quality articles which can be used for websites, blogs, or for newsletter campaign
**Topic 35. Pharmaceutics** ADC Therapeutics is a clinical-stage oncology drug discovery and development company focused on advancing highly potent and targeted antibody drug conjugates (ADCs) for the treatment of hematological cancers and solid tumors. Our ADCs are developed using the latest-generation pyrrolobenzodiazepine (PBD) dimer technology providing a superior therapeutic index compared to first-generation PBD ADCs in preclinical studies. PBD dimers are very potent toxins that actively kill cancer cells and have a differentiated mechanism of action than warheads commonly used in other ADCs
**Topic 36. Beauty and cosmetics** Body & Soul offers natural products for bath, body, hair, aromatherapy, massage, and more. It offers Shea, a body butter with natural sun protection; rich fatty acids; and vitamins A, E, and F. It also helps in dead skin renewal, maintaining radiant and dewy results. Shea comes infused with a range of botanicals such as chamomile, cucumber, and aloe vera
**Topic 37. Legal and professional services (p-KIBS)** Are you starting up a new business and need a legal law firm for creating your legal documents like company registration, copyright registration, trademark registration, and LLP agreements, then try out Evaluer. It is the top leading legal service provider in India that offers quality services at affordable prices
**Topic 38. Parking** Park24 × 7 is a platform to find and book perfect space to park a car. They connect drivers in search of parking with anyone who has a space going spare, whether in a car park, private driveway, and parking garage. Drivers can even park at unused parking spaces of hotels, hospitals, stores, pubs, schools, and public parking spaces

**Table 5:** Results of the regression analysis for the STM model

	Intercept	Year	Location_NA	Location_Eur	Location_Asia	Location_SA	Location_Oceania	Location_Afr
Topic 1	− 1.9270***	0.0010***	0.0023***	− 0.0020***	− 0.0035***	− 0.0050***	− 0.0013	-0.0068***
Topic 2	− 0.8387***	0.0004***	− 0.0124***	0.0070***	0.0005	− 0.0078***	− 0.0046**	0.0077***
Topic 3	− 6.5148***	0.0032***	0.0244***	0.0172***	0.0163***	0.0107***	0.0138***	0.0040**
Topic4	1.2270***	− 0.0006***	− 0.0087***	− 0.0092***	− 0.0092***	− 0.0119***	− 0.0087***	-0.0138***
Topic 5	− 3.0655***	0.0015***	0.0047***	0.0032***	− 0.0093***	0.0063***	0.0063***	-0.0091***
Topic 6	− 1.3660***	0.0007***	0.0130***	0.0018***	0.0037***	0.0049***	0.0037**	0.0071***
Topic 7	0.1779	− 0.0001	− 0.0055***	− 0.0085***	− 0.0010	0.0057***	− 0.0046**	− 0.0008
Topic 8	− 0.5023***	0.0003***	0.0012***	− 0.0022***	− 0.0035***	− 0.0025***	− 0.0016	-0.0040***
Topic 9	1.6540***	− 0.0008***	− 0.0030***	0.0010**	0.0291***	0.0036***	− 0.0002	− 0.0026**
Topic 10	0.1938*	− 0.0001*	0.0068***	0.0065***	0.0022***	0.0012	0.0064***	0.0072***
Topic 11	0.9653***	− 0.0005***	− 0.0128***	− 0.0110***	− 0.0066***	− 0.0197***	− 0.0124***	-0.0113***
Topic 12	1.4820***	− 0.0007***	− 0.0008	0.0209***	0.0136***	0.0038***	0.0059***	0.0051***
Topic 13	− 1.0210***	0.0005***	0.0024***	0.0008	0.0027***	− 0.0003	0.0014	0.0007
Topic 14	− 1.3010***	0.0007***	0.0071***	0.0108***	0.0059***	0.0101***	0.0070***	0.0050***
Topic 15	− 1.5910***	0.0008***	− 0.0020***	− 0.0033***	− 0.0036***	− 0.0013	0.0021	0.0004
Topic 16	0.0447	0.00001	0.0014***	0.0015***	0.0032***	0.0005	0.0023*	0.0034**
Topic 17	− 2.3380***	0.0012***	− 0.0103***	− 0.0082***	− 0.0110***	− 0.0158***	− 0.0006	-0.0120***
Topic 18	− 6.4833***	0.0032***	0.0027***	0.0086***	0.0042***	0.0114***	0.0094***	0.0245***
Topic 19	− 1.3560***	0.0007***	0.0091***	0.0061***	0.0016*	− 0.0030**	0.0072***	− 0.0022
Topic 20	− 0.1491	0.0001	0.0103***	0.0089***	0.0077***	0.0005	0.0035**	− 0.0004
Topic 21	1.1900***	− 0.0006***	0.0005	0.0004	− 0.0016***	− 0.0025***	− 0.0023*	− 0.0016
Topic 22	0.6145***	− 0.0003***	0.0123***	0.0128***	0.0099***	0.0166***	0.0136***	0.0018
Topic 23	− 1.3360***	0.0007***	0.0040***	0.0061***	0.0026***	0.0116***	0.0064***	0.0483***
Topic 24	0.5180***	− 0.0002***	0.0118***	0.0091***	0.0090***	− 0.0039**	0.0132***	0.0151***
Topic 25	3.7150***	− 0.0018***	− 0.0096***	− 0.0065***	− 0.0035***	− 0.0098***	− 0.0114***	-0.0074***
Topic 26	− 1.6350***	0.0008***	0.0025***	0.0030***	0.0030***	0.0003	0,0029***	-0.0017*
Topic 27	1.2990***	− 0.0006***	0.0057***	0.0087***	0.0047***	− 0.0028**	0.0040**	-0.0004
Topic 28	0.8556***	− 0.0004***	− 0.0053***	− 0.0063***	− 0.0106***	− 0.0060***	− 0.0088***	-0.0087***
Topic 29	1.7240***	− 0.0008***	− 0.0005	− 0.0033***	− 0.0047***	− 0.0043***	− 0.0055***	− 0.0036**
Topic 30	3.5060***	− 0.0017***	0.0226***	− 0.0010*	− 0.0142***	0.0706***	− 0.0045***	0.0147***
Topic 31	1.1820***	− 0.0006***	− 0.0139***	− 0.0119***	− 0.0065***	0.0021	− 0.0070***	− 0.0012
Topic 32	2.7960***	− 0.0014***	− 0.0131***	− 0.0159***	− 0.0196***	− 0.0062***	− 0.0188***	-0.0162***
Topic 33	4.8400***	− 0.0024***	− 0.0210***	− 0.0172***	− 0.0087***	− 0.0165***	− 0.0167***	-0.0151***
Topic 34	3.7270***	− 0.0018***	− 0.0314***	− 0.0323***	− 0.0290***	− 0.0299***	− 0.0335***	-0,0263***
Topic 35	− 1.0080***	0.0005***	0.0201***	0.0152***	0.0082***	0.0010	0.0072***	− 0.0007
Topic 36	− 0.1075	0.0001	− 0.0028***	− 0.0034***	− 0.0010**	− 0.0044***	− 0.0028***	-0.0054***
Topic 37	2.4868***	− 0.0012***	− 0.0128***	− 0.0087***	0.0192***	− 0.0082***	0.0276***	0.0050***
Topic 38	− 0.6506***	0.0003***	0.0012***	0.0014***	− 0.0001	0.0012***	0.0014***	0.0014***

Note: ***, **, and * denote 0.1%, 1%, and 5% significance level, respectively. Coefficients indicate whether prevalence of respective topics changes with the value of the covariates.

## Footnotes

1 https://about.crunchbase.com/about-us/

2 https://support.crunchbase.com/hc/en-us/articles/360043146954-What-Industries-are-included-in-Crunchbase-

3 We use Crunchbase region classification. For more details, see https://support.crunchbase.com/hc/en-us/articles/360010018153-How-are-regions-in-Crunchbase-defined-

4 We selected 50 as an upper bound to ease overview and comparison of different topics. Another argument supporting our choice is that the original Crunchbase classification has 46 topics that entrepreneurs can choose from. Therefore, we were aiming to improve the classification by maintaining a comparable number of topics.

5 The statements are chosen from the ten startup descriptions with the highest topic prevalence.

6 Henceforth, we refer to topic #X as T#X.

7 As a robustness test, we also tried a 10% threshold. While the absolute number of topics naturally falls with this alternative threshold value, this does not affect our conclusions concerning the dynamics of the topic number per startup.

8 Note that 12.1% of all startups in the Crunchbase database have no region they assigned to, which allowed us to include dummy variables for each of the regions.

9 For a full set of regression results, see Table 5 in Appendix 2.

## Code availability

The custom R code used can be provided on request.
